# Crosstalk between Thyroid Carcinoma and Tumor-Correlated Immune Cells in the Tumor Microenvironment

**DOI:** 10.3390/cancers15102863

**Published:** 2023-05-22

**Authors:** Mingyuan Song, Qi Liu, Wei Sun, Hao Zhang

**Affiliations:** Department of Thyroid Surgery, The First Hospital of China Medical University, 155 Nanjing North Street, Shenyang 110001, China; 2022120781@cmu.edu.cn (M.S.);

**Keywords:** thyroid carcinoma, immune cell, tumor microenvironment, immunotherapy, crosstalk

## Abstract

**Simple Summary:**

The majority of DTCs exhibit a favorable prognosis, while a minority of subtypes are fatal, thus highlighting the need for effective treatments for aggressive TCs. Molecular studies of this aggressive tumor have received increasing attention. Recent studies have revealed a crosstalk between immune cells and TC in TME, emphasizing the role of chemo-kinesin/cytokines. Immunotherapy has emerged as a promising avenue to combat aggressive TC, with studies highlighting the mechanisms underlying TC progression, identifying immune cells as prognostic markers and therapeutic targets, and recommending immunotherapy-based interventions. However, the development of highly specific and safe targeted drugs for TC remains a major challenge and requires a more detailed understanding of the molecular underpinnings and immunotherapy.

**Abstract:**

Thyroid cancer (TC) is the most common malignancy in the endocrine system. Although most TC can achieve a desirable prognosis, some refractory thyroid carcinomas, including radioiodine-refractory differentiated thyroid cancer, as well as anaplastic thyroid carcinoma, face a myriad of difficulties in clinical treatment. These types of tumors contribute to the majority of TC deaths due to limited initial therapy, recurrence, and metastasis of the tumor and tumor resistance to current clinically targeted drugs, which ultimately lead to treatment failure. At present, a growing number of studies have demonstrated crosstalk between TC and tumor-associated immune cells, which affects tumor deterioration and metastasis through distinct signal transduction or receptor activation. Current immunotherapy focuses primarily on cutting off the interaction between tumor cells and immune cells. Since the advent of immunotherapy, scholars have discovered targets for TC immunotherapy, which also provides new strategies for TC treatment. This review methodically and intensively summarizes the current understanding and mechanism of the crosstalk between distinct types of TC and immune cells, as well as potential immunotherapy strategies and clinical research results in the area of the tumor immune microenvironment. We aim to explore the current research advances to formulate better individualized treatment strategies for TC patients and to provide clues and references for the study of potential immune checkpoints and the development of immunotherapy technologies.

## 1. Introduction

In recent years, the incidence rate of thyroid carcinoma (TC) has increased 3% annually. In addition, its incidence rate accounts for 2.1% of all cancer diagnoses worldwide. Moreover, the growth in the incidence of TC worldwide is mainly driven by the increase in papillary thyroid carcinoma (PTC) [1,2,3]. PTC is the most prevalent histological subtype (89.1%) and is called “lazy carcinoma” due to its positive prognosis. Compared to PTC, follicular thyroid carcinoma (FTC), which is also a differentiated thyroid carcinoma (DTC), has a relatively poorer prognosis. Anaplastic thyroid carcinoma (ATC) is a rare malignant tumor, accounting for 1–2% of all TCs, yet it is the predominant cause of TC death [3,4]. Despite the fact that the prognosis of most TC is satisfactory, some types of thyroid carcinoma are associated with high mortality, including radioiodine-refractory differentiated thyroid cancer (RR-DTC) and ATC. They face a variety of challenges in clinical treatment [4,5]. Routine post-operative treatments for the above types of TC currently include thyrotropin, iodine 131, etc. Nonetheless, these techniques have certain limitations. Radiotherapy can have some irreversible side effects on patients, namely an augment in the incidence rate of a second primary carcinoma [6,7]. Moreover, targeted therapy for recurrent RR-DTC and ATC is still unable to attain satisfactory results [8,9].

The resistance of tumor cells to immune destruction is a defining feature of cancer. Tumor microenvironment (TME) refers to the complex environment required for tumor cells to survive and develop, and is composed of tumor cells and their surrounding immune cells, inflammatory cells, fibroblasts, various signal molecules, extracellular matrix, and surrounding blood vessels. Tumor cells and their microenvironment constantly interact to influence tumor growth. In the TME, pro-inflammatory cytokines and chemokines secreted by immune cells are activated by tumors to propel tumor cell proliferation, metastasis, and dedifferentiation [10], whereas in healthy people, immune cells can kill tumor cells. However, with the establishment of TME, various immune cells enhance the immunosuppressive capacity of tumor cells, thereby preventing the destruction of innate immunity [11]. These immune cells are highly expressed in TC and chiefly distributed in and around the tumor. Moreover, inflammatory factors secreted by immune cells or tumor cells may contribute to tumor progression and invasion [12].

**Figure 1:**
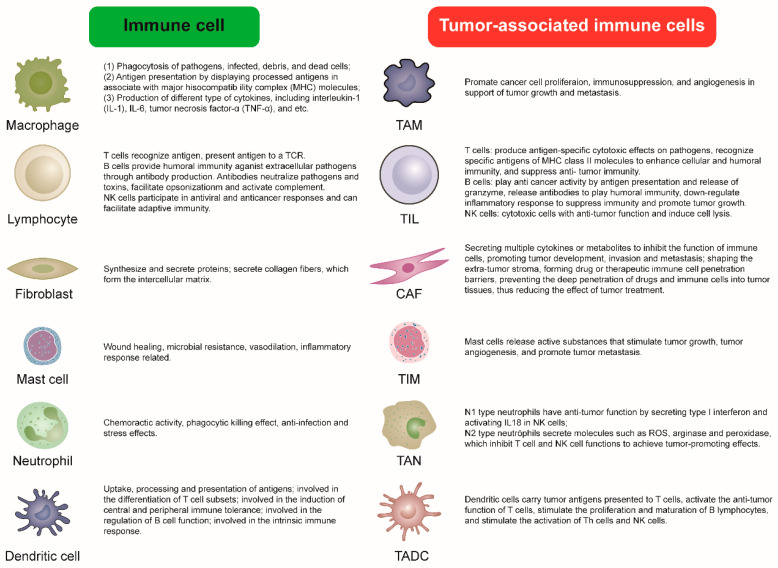
The functional differences between normal immune cells and tumor-associated immune cells.

In the American Thyroid Association’s 2021 annual report, it was proposed that the interaction between TC and its microenvironment should receive heightened attention [13]. Tumor cells and immune cells in the TME form a crosstalk mechanism linked by metabolites. By recruiting immune-suppressing cells, tumors can lessen their immunogenicity. To evade surveillance, tumors can also damage the host immune cells in the TME via additional immunosuppressive mechanisms [10]. The tumor or microenvironment will likewise change the immune response mode of the tumor [14]. Additionally, the correlation between immune cell infiltration and thyroid cell DNA repair gene disorder exemplifies the effect of tumor immunity on TC progress [15]. Consequently, the proliferation, metastasis, and dedifferentiation of TC cells will be influenced by immune cells, but tumor cells will also act on immune cells by activating, polarizing, and reprogramming them. This crosstalk between tumor cells and immune cells has become a hotspot for a great deal of research and one potential immunotherapeutic strategy. The research on tumor-associated immune cells not only aids in investigating the occurrence of TC and explains the mechanism of tumor invasion and metastasis, but also aids in the creation of innovative and effective immunotherapy techniques in the individualized treatment of TC. Therefore, we systematically and comprehensively review the crosstalk between TC and tumor-correlated immune cells, as well as novel ideas for the development of immunotherapy techniques. The functional differences between normal immune cells and tumor-associated immune cells are illustrated in Figure 1.

## 2. Crosstalk between Thyroid Carcinoma and Tumor-Associated Macrophages

Tumor-associated macrophages (TAMs) are a major type of tumor-infiltrating immune cell. TAMs are divided into two subtypes based on their function: classically activated M1 macrophages and alternatively activated M2 macrophages [16]. The former has typical anti-tumor functions, including direct cytotoxicity and antibody-dependent cell-mediated cytotoxicity. The latter facilitates the production and metastasis of tumor cells and impedes the anti-tumor immune response mediated by T cells, consequently promoting tumor angiogenesis and further triggering tumor deterioration [17]. Both M1 and M2 macrophages are highly plastic, and they are able to transform into each other when TME or treatment changes [18]. In the TME, the expression of M1 markers MHC-II and CD86 is not affected by the presence of tumor cells, while the expression of M2-related markers CD206, CD163, and MerTK are markedly up-regulated in TC. M2-type macrophages can facilitate tumor activity [19]. Notably, M2 macrophages can be divided into M2a (induced by IL-4 or IL-13), M2b (combined with IL-1β or LPS by immune complex), M2c (induced by IL-10, TGF β, or glucocorticoid induction), and M2d (conventional M2 macrophages that play an immunosuppressive role) [20,21,22]. TAMs can influence tumor cell activity, metastasis, and immunosuppression [23]. These polarized TAMs contribute significantly to the crosstalk between TME and TC.

### 2.1. Cytokine/Chemokine Mediated Crosstalk in Tumor Microenvironment

Immune cells secrete substances called cytokines, which have an effect on other cells. Chemokines, also known as chemotactic cytokines, are one of the superfamilies of cytokines and have directional chemotaxis. Both cytokines and chemokines play a crucial role in TME. As mediators between TAMs and tumor cells, they effectively facilitate tumor deterioration and tumor angiogenesis and remarkably induce macrophages to transform into M2 [24]. In other endocrine tumors, including pancreatic carcinoma, TAMs originating from embryonic hematopoietic cells serve as one of the paramount factors triggering tumor metastasis [25]. Similarly, TAMs have a similar effect on endocrine tumors of thyroid carcinoma. As the initial treatment option following DTC surgery, TSH inhibition is one of the most effective means of preventing tumor recurrence. As confirmed in a study conducted by Song et al., TSH causes the release of VEGF-A via the PI3K/AKT/mTOR or ERK signal pathway, which enhances the expression of CD31, CD163, and CXCL8, thus reinforcing the infiltration of macrophages and promoting tumor angiogenesis and proliferation. As verified in clinical data, patients with high expression of VEGF-A have higher distant metastasis rates and lower disease-specific survival rates [26]. CXCL8, which originates from TAMs, is one of the highest expressed cytokines in the TME of PTC. CXCL8 can facilitate PTC cell transfer through the CXCR1/2 axis [27]. Moreover, this paracrine action can also polarize TAMs toward M2 to some degree [28]. Consequently, the crosstalk mediated by CXCL8 is crucial in the formation of TME of PTC. Targeting CXCL8 may be one of the potential strategies for immunotherapy [29]. Likewise, CXCL16 is also a specific cytokine secreted in the TME of PTC, since it is secreted in minute amounts by individual PTC cell lines or TAMs [30]. Under the stimulation of a conditioned medium co-cultured with PTC and TAMs, the CD163, IL-10, and CD206 of TAMs are up-regulated, and in PTC, CXCR6, a receptor for CXCL16, is activated simultaneously to increase the expression of angiogenesis-related genes (PGF and EGF) and BRAF genes, thereby promoting PTC cell invasion and lymph node metastasis. As a consequence, CXCL16 mediates the crosstalk between PTC and TAMs, and targeted inhibition CXCL16 may be a novel treatment for advanced thyroid carcinoma [31].

The diffuse sclerosis variant of papillary thyroid carcinoma is a unique type of PTC, featured by the extensive invasion of lymphatic vessels and blood vessels by tumor cells [32]. In comparison to classic PTC, TAMs are abundant in infiltrating lymphocytes of diffuse sclerosis variants, and Decoy receptor 3 (DcR3) is overexpressed in a variety of tumors and can facilitate tumor angiogenesis, dendritic cell apoptosis, and regulate the function of TAMs [33]. As a result, DcR3 can propel TAMs to upregulate the membrane surface protein CD163 which induces macrophages to transform into M2. Consequently, DcR3 is critical in the crosstalk of PTC in TAMs [32,34]. M2-TAMs propel the production of IL-6 in PTC and induce the increment of PD-L1 expression by activating the synergistic effect of MAPK and JAK-STAT3 signaling pathways, and finally strengthening the invasion ability of TC [35]. Similarly, by activating the AKT/mTOR-dependent glycolysis pathway, PTC-produced lactic acid increases the production of IL-6, which results in the reprogramming of TAMs [36]. IL-6 is thus the positive recurrent link between PTC and TMEs.

Furthermore, FTC can secrete the CCL15 chemokine to recruit TAMs and construct a tumor microenvironment, while CCL15 expression is low in thyroid adenoma. Consequently, in the TME of FTC, the mechanism by which CCL15 recruits TAMs is central to the research and development of FTC drugs [37]. As has been forcefully proven by existing relevant studies, monocyte chemoattractant protein-1 (MCP-1) in PTC cells, as an attractant chemokine for monocytes, participates in the tumor immune response [38]. In clinical research, Tanaka et al. noticed that the expression level of MCP-1 was positively associated with tumor size and lymph node involvement. Hence, MCP-1 expression promotes tumor invasion and is an independent predictor of PTC recurrence [39]. This exploration also demonstrates that MCP-1 mediates the crosstalk between tumor cells and TAMs in TME.

### 2.2. Roles of Tumor-Associated Macrophages in the Tumor Microenvironment of Thyroid Carcinoma

TAMs facilitate the escape of thyroid carcinoma from the TME. As demonstrated by Qing et al., the density of TAMs in PTC is noticeably higher in comparison with benign thyroid disease, and M2 is the principal macrophage phenotype. Furthermore, they have found evidence that the polarization of TAMs towards M2 enhances the ability of tumor cells to metastasize and invade lymph nodes [40]. Reactive oxygen species (ROS) are widely believed to cause cell damage, but ROS also play a crucial part in the differentiation of M2 macrophages and TAMs. Moreover, Rabbold et al. observed that TAMs can heighten the synthesis of lipids, thereby inducing the production of ROS. Stimulating toll-like receptor 4 through lipopolysaccharide can augment the expression of cytokines, such as TNF-α, IL-6, and IL-10, and exacerbate TAMs to polarize toward M2, thus affecting tumor cell invasion and angiogenesis [19]. Prostaglandin-endoperoxide synthase in PTC can also promote the secretion of PGE2 and polarize TAMs toward the M2 subtype [41]. BRAF is a cytoplasmic serine-threonine protein kinase, its mutation rate is high in TC [42], and the mutation rates are primarily associated with the recurrence and heightened mortality of TC [43,44]. After conditional activation of BRAF (V600E) in mouse models, the expression of CSF-1 cytokine secreted by TAMs was heightened, which resulted in the recruitment of TAMs by PTC and the transformation of TAMs into M2 type, thereby advancing the development of PTC. Notably, TAMs are a potential therapeutic target for patients with advanced TC [45]. Through in vitro experiments, LV et al. indicated that M2 TAMs in PTC can also act on the Wnt/β-catenin pathway by increasing the secretion of Wnt1 and Wnt3a, thereby promoting the dedifferentiation, proliferation, and metastasis of PTC [46]. Furthermore, TNF-α that originates from TAMs in PTC can propel the expression of IL-32α and IL-32β. Moreover, overexpression of IL-32β increases the expression of IL-8, which is advantageous for the survival of TC cells [47].

CD68 is a TAM marker, and is overexpressed in poorly differentiated thyroid carcinoma (PDTC) and ATC compared to benign or normal thyroid tissues. The higher the malignancy of the tumor, the higher the expression of CD68. In addition to that, the expression of CD68 in PDTC is associated with lymph node metastasis and distant metastasis of carcinoma [48]. In ATC, an increase in TAM content is accompanied by a decrease in survival rate. The elevation of CXCR4 expression will trigger an increase in CD163-positive TAM density [49]. Additionally, TAMs expressing CD68 and CD163 infiltrate ATC cells and produce CD47 to assist tumors in escaping the surveillance of immune cells [50]. As revealed by Zhu et al., methyltransferase-like 3 (METTL3) enhances the expression of STEAP2 through an m6A–YTHDF1-dependent mechanism and then inhibits PTC proliferation and metastasis by inhibiting the Hedgehog signaling pathway and EMT [51]. Nonetheless, a separate study on METTL3 demonstrated that METTL3 expression increases miR-222-3p expression by accelerating the m6A modification of pri-miR-222-3p. In this case, the overexpression of miR-222-3p decreases the expression of serine/threonine stress kinase 4 and enhances the ability of PTC cells to proliferate, form colonies, migrate, and invade, which results in a poor prognosis for patients [52]. Another animal experiment demonstrated that the METTL3/MALAT1/PTBP1/USP8/TAK1 axis can also increase TAM polarization towards M1 [53]. Currently, the effect of METTL3 on TC is controversial, and the mechanism must be further investigated.

In conclusion, carcinoma tissue can recruit a large number of TAMs in TME, which can significantly impact the prognosis of thyroid carcinoma. Cytokines released by tumors can alter the TAM subtype, thereby promoting or inhibiting carcinogenesis.

## 3. Interaction between Thyroid Carcinoma and Tumor Infiltrating Lymphocytes

The term “tumor-infiltrating lymphocytes” (TILs) refers to white blood cells recruited from blood circulation by tumor tissue. Massive TIL infiltrates indicate that an anti-tumor immune response is taking place in the body [54]. The infiltration of TILs is closely associated with tumor progression. TILs include T cells, B cells, and natural killer (NK) cells. Furthermore, T lymphocytes are classified in accordance with their receptor subunits and their core lineages are labeled CD8 and CD4. The infiltration of CD8+T cells is associated with an increase in the rate of disease-free survival, and the infiltration of CD8+T cells and CD4+T cells is positively associated with the reduction of tumor sizes [14]. Tumor-infiltrating B lymphocytes, including B cells and plasma cells, are also involved in the anti-tumor immune response. Tumor-infiltrating B lymphocytes facilitate the anti-tumor immune response by presenting antigenicity to T cells [55]. NK cells are a paramount part of the innate immune system. NK cells serve as killer cells without prior sensitization and contribute to the formation and metastasis of tumors through the production of T helper cell type 1 or cytokines, growth factors, and chemokines [56].

### 3.1. Interaction between Tumor Infiltrating Lymphocytes and Papillary Thyroid Carcinoma

Indoleamine 2,3-dioxygenase (IDO) has been demonstrated to be overexpressed in a myriad of carcinomas and associated with T lymphocytes in TMEs [57]. IDO inhibitors can simultaneously be used as a drug to improve the therapeutic effect of carcinoma [58,59]. The expression of IDO is high in thyroid micropapillary carcinoma and the expression of IDO is significantly correlated with the decline of CD3+TIL and the augmentation of FOXP3+TIL. In particular, promoting the increment of FOXP3+TIL, lessening the infiltration of CD3+T, and weakening the immune response are associated with enhanced invasion of thyroid micropapillary carcinoma [60]. Triggering the receptor expressed on myeloid cells (TREM), which belongs to the immunoglobulin superfamily that is expressed by bone marrow cells, plays a key role in immune response [61]. In addition, the expression of TREM1 in PTC is heightened and correlated with BRAF. Thus, hypomethylation of TREM1 will increase the recurrence of PTC. Overexpression of TREM in regulatory T cells (Tregs) leads to increased PTC infiltration, which in turn promotes the progression of PTC [62]. Through in vitro experiments, French et al. illustrated that Tregs are enriched in the metastatic lymph nodes of PTC patients, indicating that the recruitment of Tregs is part of the invasiveness of PTC [63].

Transmembrane protein receptor PD-1, also known as CD279, is associated with programmed cell death [64]. PD-L1, also recognized as CD274, is one of the ligands of PD-1 [65]. Both of them are involved in the maintenance of immune homeostasis. Blocking PD-1 signaling can significantly increase the tumor’s immune evasion ability [66]. As demonstrated by previous studies, carcinoma can inhibit the host immune response by promoting the combination of PD-1 and PD-L1 [67]. Notably, PD-1’s ability to transmit inhibitory signals effectively promotes the development and function of Tregs and inhibits autoimmunity [68]. As French’s team also demonstrated, the increased expression of Tregs and PD-1 (+) T cells in PTC lymphocytes elevate the probability of tumor invasion and recurrence probability [69]. According to an experiment conducted by Wang et al., the levels of PD-L1 and PD-1 are strikingly heightened in children with PTC. Additionally, by connecting with PD-1 expressed on the surface of CD8+T cells, extract vascular programmed death-ligand 1 could inhibit the activation of CD8+T cells and reduce tumor immunity [70]. CXCR5+/CD8+ T cells have been shown to correlate with a good prognosis of gastric carcinoma and liver carcinoma [71,72]. Zhou et al. noticed that when CD8+T cells in PTC were infiltrated by CXCR5, the expression of PD-1 was heightened. Furthermore, an increase of a series of cytokines (including IL-2, IFN-γ, and TNF-α) promotes lymphocyte proliferation and subsequently affects tumor deterioration [73]. TILs can impel tumor deterioration through the expression of IDO and PD-1, and corresponding research should investigate additional therapeutic targets.

Cyclo-oxygenase-2(COX-2) is a crucial component of the inflammatory response and is also involved in the deterioration of a myriad of carcinomas. The elevated expression of COX-2 facilitates tumor invasion and angiogenesis and is linked to lymph node metastasis [74]. Thereafter, the expression of COX-2 in PTC is affected by hepatocyte growth factor and Met protein. The elevated expression of COX-2 results in an augmented invasion of tumor cells and lymph node metastasis [75,76]. Moreover, COX-2 inhibits tumor immunity by reducing the cytolytic activity of CD8+ T cells, which results in a poor prognosis for DTC patients [77]. Indoleamine 2,3-dioxygenase 1 (IDO1) is an immunosuppressive enzyme which can degrade tryptophan, and the expression of IDO1 is closely related to COX-2 [78]. By reducing the expression of constitutive IDO1, COX-2 inhibitors can enhance the efficacy of carcinoma immunotherapy strategies [79].

Apolipoprotein E (APOE) is a kind of apolipoprotein produced in diverse tissues, which can form lipoproteins by combining with lipids, and transport them to various tissues and organs of the body. It is commonly believed to be linked to Alzheimer’s disease and atherosclerosis [80]. In animal experiments, Tavazoie et al. have proved that the LXR/APOE axis participates in immune response and enhances anti-tumor ability by enhancing lymphocyte activation [81]. According to the most recent study by Lin et al., APOE could increase the infiltration of B cells, CD8+T cells, and other immune cells [82]. Furthermore, Huang et al. discovered that fat mass and affinity-associated protein (FTO) could inhibit m6A methylation of APOE and subsequently hinder the glycolysis of PTC through the IL-6/JAK2/STAT3 signal pathway to inhibit tumor growth [83]. Zheng et al. noticed that the high expression of AHNAK nucleoprotein 2 in PTC reduced the infiltration of CD8+T cells, whereas the infiltration of Tregs was increased [84]. In addition, the density of TILs is increased in PTC with the BRAF V600E mutation [85], while the infiltration of CD8+ T cells is reduced [86]. These changes ultimately drive the progression of PTC and further affect patient outcomes.

As the first congenital lymphoid cells to be identified, NK cells can kill cells and produce proinflammatory factors [87]. NK cells are usually classed into two subpopulations based on the expression of CD56 and CD16 on the cell membrane surface [88]. The most representative NK cell subsets are CD56brightCD16− and CD56dimCD16+ populations, which are associated with most functions of NK cells [89]. CD56bright and CD56dim have the functions of producing cytokines and cytolytics, respectively [90]. Through in vitro experiments, Gogali et al. determined that the infiltration of CD3−CD16+CD56dim NK cells was positively correlated with the progress of PTC, and the infiltration of CD3−CD16−CD56bright NK cells showed a negative correlation with the incidence and development of PTC [91]. These results supported the correlation between TILs and PTCs.

### 3.2. Interaction between Tumor Infiltrating Lymphocytes in Follicular Thyroid Carcinoma

It should be noted that the TILs in FTC play the same role in PTC. Arif et al. noticed during an in vitro study that interstitial lymphocytes infiltrated extensively in FTC [92]. PD-L1, produced by tumor cells and tumor-correlated immune cells, takes part in the progression of numerous carcinomas. Current research indicates that PD-L1 suppresses the anti-tumor immune response by inhibiting the activation of T cells, which is the most common blocking target of immune checkpoints [93]. Saburi et al. noticed that PD-L1 was highly expressed in FTC, and its expression was higher in the invasive edge of the tumor and the infiltrating tumor-correlated immune cells than in the tumor center, which may be linked to the high concentration of T cells in this region [94]. Anti-PD-1 therapy has become one of the most effective treatments for advanced TC [95]. As a marker of primary Tregs in carcinoma and autoimmune diseases, FOXP3 is expressed in activated T cells. FOXP3+ T cells express low levels of cytokines in the tumor microenvironment, which contributes to tumor immune evasion [96]. Chu et al. have proved that Foxp3 had increased expression in PTC and FTC and promoted cell proliferation and migration. Inhibition of Foxp3 leads to a rise in expression and activity of PPARγ, as well as lowering the expression of NF-κB and Cyclin D1, which inhibit tumor growth and migration and promote cell apoptosis. This provides a new targeted treatment option for thyroid carcinoma [97]. Moreover, IDO1 is an enzyme that catalyzes the oxidative cleavage of tryptophan. Its decrease stimulates the proliferation of effector T lymphocytes and the maturation of Tregs [98]. A cellular experiment implemented by Moretti revealed that IDO1 is expressed primarily in thyroid carcinoma tissues and more highly expressed in invasive types. Furthermore, co-culture experiments indicated that the expression of IDO1 inhibited the proliferation of activated T lymphocytes in the tumor microenvironment, consequently affecting the development and occurrence of tumors [99].

### 3.3. Interaction between Tumor Infiltrating Lymphocytes in Anaplastic Thyroid Carcinoma

TILs also play an important role in ATC. The IFNγ-JAK2 signaling pathway in ATC promotes tumor metastasis by regulating the augmented expression of ICAM1 and PD-L1 [100]. Moreover, Wang found that the expression of UCA1 was heightened in ATC, which can propel the expression of PD-L1 by targeting miR-148a and diminishing the killing ability of CD8+T cells and the secretion of cytokines, before eventually leading to the weakness of the tumor immune response [101].

Lymphocyte-to-monocyte ratio (LMR), defined as the absolute lymphocyte count divided by the absolute monocyte count, NK cell likewise affects the progress of TC. Current studies show that LMR correlates with the prognosis of multiple carcinomas [102]. As indicated by Ahn et al.’s study, among those patients with radioiodine refractory differentiated thyroid carcinoma considered with sorafenib, patients with high LMR have higher overall survival (OS) and progression-free survival (PFS) than patients with low LMR. LMR may therefore be a prognostic biomarker for patients with radioiodine-resistant differentiated thyroid carcinoma [103].

Apart from that, LMR can reflect host immune function and TAM infiltration in ATC. Low-LMR patients have a low survival rate [104]. NK cells, which are effector lymphocytes in the innate immune system, can promote the development of anti-tumor response [105]. PGE2 was found to interfere with the aggregation of immune cells in tumors by blocking the early activation of tumor-derived NK cells [106]. In comparison with PTC, Park et al. noticed that PGE2 was highly expressed in ATC. PGE2 secreted by tumor cells inhibits the expression of NK cell-activated receptors by blocking EP2 and EP4 receptors on the surface of NK cells. Simultaneously, PGE2 inhibits the functional maturation and cytotoxicity of NK cells [107]. The fundamental experiment conducted by Wennerberg et al. demonstrated that ATC cells were sensitive to the cleavage of NKG2D-positive NK cells, and the ATC cells could attract CXCR3-positive NK cells. PGE2 can inhibit COX2-positive ATC from recruiting NK cells [108]. These experiments demonstrate that PGE2 can accelerate the progression of TC by inhibiting NK cells, and PGE2 can be used as a new therapeutic target for TC patients.

## 4. Crosstalk between Thyroid Carcinoma and Cancer-Associated Fibroblasts

Generally speaking, cancer-associated fibroblasts (CAFs) are defined as fibroblasts inside or around tumors. CAFs exert a paramount influence on the construction as well as the remodeling of the extracellular matrix and play a vital part in the metabolism and immune reprogramming of the tumor microenvironment. CAFs also can secrete several chemokines that promote tumor invasion and metastasis and the occurrence of anti-tumor immunity [109], as well as enhance tumor chemotherapy resistance in tumors [110]. Nevertheless, CAFs can also be negative regulators in tumor development [111]. Due to this, immunotherapy-targeted CAFs have become a hot topic in carcinoma treatment [112,113].

### 4.1. Crosstalk between Cancer-Associated Fibroblasts and Thyroid Carcinoma

By carrying out their immunohistochemical study, Cho et al. discovered that CAFs were widely present in PTC. In addition, a multivariate analysis revealed that CAFs were independent risk factors for lymph node metastasis in PTC. Hence, CAFs can be used as predictive markers of lymph node metastasis in PTC patients [114]. Wen et al. noticed that the density of CAFs and the degree of differentiation of thyroid carcinoma were correlated with a poor prognosis [115]. By carrying out in vivo experiments, Saitoh et al. determined that CAFs facilitated the growth of thyroid carcinoma cells in vivo and in vitro in the thyroid gland. On this basis, they hypothesized that this facilitation was due to the release of soluble factors [116]. Fozzatti et al. provided an additional explanation for the above speculation. By co-culturing ATC cells with CAFs in vitro, they noticed that CAF-conditioned medium could facilitate the proliferation and invasion of thyroid carcinoma cells. They also discovered that this effect could propel the EMT of thyroid carcinoma cells. ROS and IL-6 are known to affect the activation and differentiation of CAFs. In the co-culture system, the secretion level of these two components was increased. As a consequence, CAFs may shift phenotype and function by secreting IL-6 and ROS and thereby increase the aggressiveness of thyroid carcinoma [117].

Fibroblast growth factor (FGF), typically secreted by endothelial cells, can facilitate the growth of CAFs. FGF has been shown to play a pivotal role in multifarious carcinomas. An abnormal FGF signal will push tumor development by promoting tumor cell proliferation and tumor angiogenesis [118]. FGF19 is a member of the FGF family of endocrine factors and has been proven to play a crucial role in a variety of carcinomas [119,120,121]. By detecting the immunohistochemical characteristics of TC patients, Zhang et al. discovered that FGF19 was highly expressed in TC tissues and was associated with malign behaviors including tumor invasion and lymph node metastasis. Accordingly, FGF19 can be employed as a molecular marker for early diagnosis and a target for therapeutic intervention [122]. Moreover, FGFR-1-4 is expressed in normal thyroid tissue and plays a part in promoting or inhibiting carcinoma progression, according to Bernard et al. [123,124]. The content of FGF21 in PTC patients is comparatively high, and FGF21 can promote tumor deterioration by activating the FGFR signal axis to upregulate EMT signal transduction. The level of FGF21 is bound up with metabolism, and FGF21 also mediates obesity in PTC patients [125]. Simultaneously, Kondo et al. have founded that FGF7 stimulates endogenous FGFR2-IIIb, which gives rise to DNA methylation and chromatin modification to decrease the expression of MAGE-A3/6, and thereby inhibits tumor growth [126]. However, research on the role of CAFs in regulating of thyroid carcinoma remains insufficient. Therefore, future research must continue to concentrate on this topic.

### 4.2. Crosstalk between Thyroid Carcinoma and Cancer-Associated Fibroblasts

Maternally expressed gene 3 (MEG3) is a chain of noncoding RNA that plays a significant role as a tumor suppressor in tumors [127]. Dadafarin et al. found that MEG3 expression level suggested infiltration of CAFs in PTC [128]. Tumor cells will secrete fibroblast activating factors through signal transduction for the purpose of driving the recruitment and activation of fibroblasts. The chemokine growth-regulated oncogene 1 (Gro-1) can induce the senescence of fibroblasts [129], and IL-6 can stimulate the activation of CAFs in prostate carcinoma [130]. In addition, ROS has been shown to promote the transformation of fibroblasts into CAFs and facilitate the activation of CAFs [131]. BRAF mutation is the most prevalent genetic alteration in TC, and are usually accompanied by increased aggressiveness. BRAF mutation can also trigger the down-regulation of tumor suppressor genes as well as the up-regulation of carcinoma-promoting molecules [132,133]. Through immunohistochemical staining, Minna et al. discovered that in BRAF-mutated TCs (including PTC, PDTC, and ATC), CAFs were enriched in the invasion edge as well as senescent TC cells. Their interaction facilitated local tumor invasion [134]. Moreover, activation of BRAF can facilitate the secretion of CAF migration factors, thus giving rise to increased CAF recruitment and the enhancement of CAF’s value-added and migration capacities [135]. All of the aforementioned findings indicate that the feasibility of developing new therapies targeted at CAFs to expand current treatment methods and improve TC patient prognoses.

## 5. Crosstalk between Thyroid Carcinoma and Other Immune Components in the Tumor Microenvironment

### 5.1. Role of Tumor-Associated Dendritic Cells in the Tumor Microenvironment of Thyroid Carcinoma

Tumor-associated dendritic cells (TADCs) are humans’ most effective antigen-presenting cells. Carcinoma cells can act on TADCs and affect the immunity of TADCs to tumors by producing cytokines. Immunotherapy related to TADCs has received increasing attention [136]. In tumor tissue, macrophage inflammatory protein-3α, serving as a chemokine, can recruit immature TADCs to the injured site and maintain the immune response by capturing antigens [137]. Tsuge et al. determined that macrophage inflammatory protein-3α was strongly expressed in PTC [138], which demonstrates that a multitude of immature TADCs accumulate in PTC. Moreover, TADCs highly express TSHα and TSHβ2, which can facilitate the proliferation and invasion of tumor cells and the occurrence of immune evasion [139]. Bergdorf discovered a correlation between the content of TADCs and the histological subtype, mutation status, T stage, and lymph node metastasis of PTC. Furthermore, the greater the concentration of TADCs, the greater the invasiveness of PTC [140]. In the future, more in-depth research is needed to explain the mechanism of TADCs in PTC progress.

### 5.2. Role of Mast Cells in the Tumor Microenvironment of Thyroid Carcinoma

Mast cells (MCs) are immune cells that originate from bone marrow and are extensively distributed in nearly all normal tissues and human carcinomas [141]. PTC with BRAF V600E mutation showed heightened MC infiltration [86], as BRAF mutation was bound up with the deterioration of thyroid carcinoma [142]. Consequently, the infiltration of MCs may hinder the development of TC. Melillo illustrated that the density of MCs in thyroid carcinoma was higher than that in normal thyroid tissue. Moreover, MCs release TNF- α, IL-6, IL-8, and other cytokines to facilitate the proliferation and invasion of carcinoma cells, thereby stimulating the development and spread of thyroid carcinoma [143]. Visciano found that MCs could stimulate Akt phosphorylation and Slug expression in TC by releasing IL-8 and maintain EMT and stemness of TC, where the density of MCs positively correlates with tumor stemness [144]. IL-8 blockade can therefore be used to treat patients with advanced TC. Finally, immunotherapy for MCs is a promising area of research.

### 5.3. Role of Tumor-Associated Neutrophils in the Tumor Microenvironment of Thyroid Carcinoma

Tumor-associated neutrophils (TANs) play a vital part in innate and adaptive immunity [145]. TANs can also affect the progress of carcinoma. However, the specific role of TANs in tumor development is still controversial [146,147]. Galdiero et al. discovered that immune cells from TC could recruit and activate TANs by producing cytokines [148]. Additionally, N6 methyl adenosine (m6A) is a ubiquitous internal modifier in eukaryotic mRNA, which can influence the metabolism and maturation of mRNA [149]. Methyltransferase such as 3 METTL3 is a methyltransferase complex which has been proven to play a paramount role in carcinoma progression [150,151]. Moreover, He et al. observed that a decrease in METTL3 could encourage PTC to produce IL-8 to recruit TANs and eventually promote PTC progress [152]. Cristinziano found that in ATC, cancer cells could produce the soluble mediators CXCL8/IL-8 and ROS to induce TANs to release neutrophil extracellular DNA traps (NETs), a network of cytosols and granulin, and promote the growth of cancer cells [153,154]. Overall, TANs can facilitate the progression of thyroid carcinoma. More importantly, inhibiting TANs is an optional method for improving the prognosis of thyroid carcinoma patients in the future.

**Figure 2:**
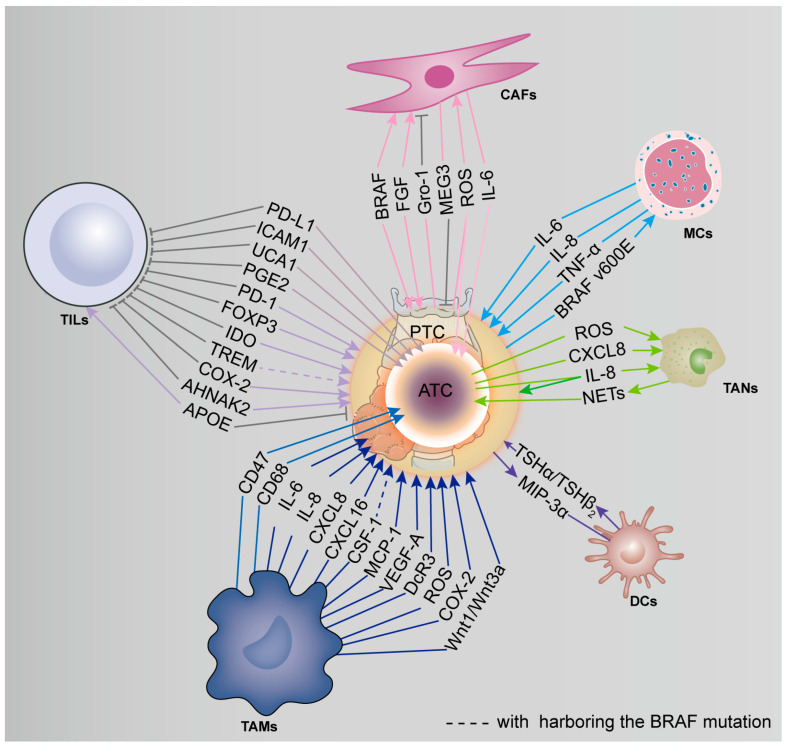
The crosstalk between tumor-associated immune cells and PTC and ATC.

The crosstalk between tumor-associated immune cells and PTC and ATC is described in Figure 2.

## 6. Potential Therapeutic Targets and Mechanisms of Thyroid Carcinoma Therapy

### 6.1. Targeted Drugs Approved in Thyroid Carcinoma

Targeted therapy for TC is mostly derived on the basis of other tumor treatments and is usually used in the treatment and research of advanced thyroid carcinoma. These targeted drugs approved by the U.S. Food and Drug Administration (FDA) for clinical immunotherapy of TC can be categorized into two parts: receptor tyrosine kinase inhibitors, which can block tumor growth signals, and tropomyosin receptor kinase inhibitors, which can block proteins required for cell growth. These approved targeted drugs are reviewed here, including the drug’s nature, dose, target, and adverse effects (Table 1).

As a potent inhibitor for multiple DTC receptor tyrosine kinases, Cabozantinib can inhibit MET, RET, and VEGFR phosphorylation as well as VEGF-induced tumor cell invasion, metastasis, and endothelial cell tube formation [155,156]. Cabozantinib has been approved for treating patients aged 12 years and older with locally terminal or metastatic DTC [157], based on the results of an international randomized double-blind trial. The study found that Cabozantinib significantly extended PFS in the intention-to-treat population compared to placebo [158]. Pralsetinib, another kinase inhibitor, can inhibit RET fusions and mutations [159] and has been indicated for the treatment of RET-mutant MTC as well as RET fusion-positive thyroid carcinoma [160]. The approval was on the basis of a multi-center, multi-queue clinical trial which demonstrated that the reaction rate of pralsetinib in patients with untreated RET-mutant MTC is 71%, whereas the overall reaction rate in patients with RET fusion-positive thyroid carcinoma receiving radioiodine treatment is 89% [161].

Lenvatinib inhibits VEGF, FGF, KIT, RET, and other growth factors, and it has anti-lymphangiogenic and anti-angiogenic activity [162]. Lenvatinib has been approved for patients with RR-DTC [163]. The approval was based on a randomized controlled trial in which Lenvatinib positively impacted remission rates and prolonged PFS in comparison to placebo [164]. Another targeted drug approved for locally relapsed or metastatic RR-DTC is sorafenib, which can inhibit VEGFR1, VEGFR2, VEGFR3, RET, and RAF [165]. A multicenter study found that the median PFS of patients treated with sorafenib was substantially longer than that of the placebo group [166].

Selpercatinib can inhibit wild-type and mutant RET as well as VEGFR1, VEGFR3, FGFR1, FGFR2, and FGFR3. Consequently, the FDA has approved it for patients with RET-mutant MTC and RET-positive thyroid carcinoma [167]. The approval was based on a multicenter, multi-queue trial that demonstrated that approximately 70% of MTC patients had an objective response to Selpercatinib and that almost all patients experienced regression of their tumors [168]. Vandetanib, an inhibitor of RET, VEGFR, and EGFR, was approved for terminal, unresectable, or metastatic MTC [169]. Furthermore, the approval was based on a randomized controlled trial. The result demonstrated obvious advantages to vandetanib in PFS, objective remission rates, disease control rates, and biochemical response [170].

Larotrectinib can competitively inhibit TRKA, TRKB, and TRKC [171], a use which has been approved in patients with NTRK mutation-positive solid tumors [172]. The trial demonstrted that Larotrectinib has a sustained anti-tumor effect in adult and pediatric patients, with PFS reaching 69% at 24 months [173]. Another drug approved for patients with solid tumors with NTRK mutations is Entrectinib [174], which can inhibit TRKA, TRKB, and TRKC, as well as ROS1 and ALK [175]. Compared to the control group, entrectinib-treated patients demonstrated a higher ORR and longer-lasting response in the test [176].

Dabrafenib and Trametinib serve as BRAF and MEK inhibitors respectively. Both have been approved for the treatment of BRAF V600E mutant locally terminal or metastatic ATC [177]. The combined treatment improves overall remission rates, duration of response and overall survival, and the toxicity is manageable [178].

Current research has made tremendous strides; however, specific targeting drugs for progressive/invasive thyroid carcinoma remain under-researched. It is unknown whether targeted drug combinations can improve efficacy and safety. In the future, more clinical trials are needed to elucidate the efficacy and adverse effects of these drugs and to verify findings in more models [179]. In addition, the feasibility of treatment and the subsequent impact of treatment remains an issue that cannot be ignored [180].

**Table 1:** Carcinoma drugs approved by the food and drug administration (FDA) for thyroid carcinoma.

Drug	FDA-Approved Indication	Mechanism of Action	Dosage	Efficacy	Mechanism of Action: Targets	Common Adverse Events	References
Cabozantinib	adult and pediatric patients 12 years of age and older with locally advanced or metastatic differentiated thyroid carcinoma	receptor tyrosine kinase inhibitor	140 mg/day	PFS: 11.0 months ORR: 18%	MET, VEGFR1, VEGFR2, VEGFR3, AXL, RET, ROS1, TYRO3, MER, KIT, TRKB, FLT3, TIE2	diarrhea, pleural effusion, pulmonary embolism, dyspnea	[157]
Pralsetinib	adult and pediatric patients ≥12 years of age with advanced or metastatic RET-mutant MTC who require systemic therapy adult and pediatric patients ≥12 years of age with advanced or metastatic RET fusion–positive thyroid carcinoma who require systemic therapy and who are radioactive iodine-refractory	receptor tyrosine kinase inhibitor	400 mg/day	ORR: 89%	DDR1, TRKC, FLT3, JAK1, JAK2, TRKA, VEGFR2, PDGFRb, FGFR1	increased AST (34%), anemia (24%), increased ALT (23%), constipation (23%), hypertension (22%)	[160]
Lenvatinib	locally recurrent or metastatic, progressive, radioactive iodine-refractory differentiated thyroid carcinoma	receptor tyrosine kinase inhibitor	24 mg/day	PFS: 18.3 months ORR: 65%	VEGFR1, VEGFR2, VEGFR3, FGFR1, FGFR2, FGFR3, FGFR4, PDGFRα, KIT,RET	Hypertension (73%), fatigue (67%), diarrhea (67%), arthralgia/myalgia (62%), decreased appetite (54%), decreased weight (51%), nausea (47%), stomatitis (41%), headache (38%), vomiting (36%)	[163]
Sorafenib	patients with progressive radioactive iodine-refractory differentiated thyroid carcinoma	receptor tyrosine kinase inhibitor	400 mg/day	PFS: 10.8 months ORR: 12.2%	RAF, VEGFR1, VEGFR2, PDGFRβ	hyperglycemia, fatigue, anemia, oral mucositis	[166]
Selpercatinib	adult and pediatric patients ≥12 years of age with advanced or metastatic RET-mutant MTC who require systemic therapy adult and pediatric patients ≥12 years of age with advanced or metastatic RET fusion–positive thyroid carcinoma who require systemic therapy and who are radioactive iodine-refractory	receptor tyrosine kinase inhibitor	160 mg twice a day	ORR: 100%	RET, VEGFR, VEGFR3, FGFR1, FGFR2, FGFR3	dry mouth, diarrhea, constipation, nausea, abdominal pain, vomiting	[167]
Vandetanib	symptomatic or progressive medullary thyroid carcinoma in patients with unresectable, locally advanced, or metastatic disease	receptor tyrosine kinase inhibitor	300mg/day	ORR: 44%	RET, EGFR, VEGFR1, VEGFR2, VEGFR3	diarrhea, hypocalcemia, asthenia, QTc prolongation, hypokalemia, keratopathy	[169]
Larotrectinib	adult and pediatric patients whose carcinomas harbor neurotrophic receptor tyrosine kinase gene fusions	tropomyosin receptor kinase inhibitor	adult and pediatric: BSA of ≥1 m^2^ s 100 mg twice daily pediatric: BSA of <1 m^2^ iss 100 mg/m^2^ twice daily	ORR: 75%	TRKA, TRKB, TRKC	increased AST level (45%), increased ALT level (45%), anemia (42%), fatigue (37%), nausea (29%), dizziness (28%), vomiting (26%), cough (26%), constipation (23%), diarrhea (22%)	[172]
Entrectinib	adult and pediatric patients whose carcinomas harbor neurotrophic receptor tyrosine kinase gene fusions	tropomyosin receptor kinase inhibitor	Pediatric: 600mg/day(BSA>1.50 m^2^), 500mg/day (BSA: 1.11–1.50 m^2^) and 400mg/day (BSA:0.91–1.10 m^2^) Adult: 600 mg/day	ORR: 57%	TRKA, TRKB, TRKC, ROS1, ALK, JAK2, TNK2	pulmonary infections, weight gain, dyspnea, fatigue/asthenia, cognitive disorders, syncope, pulmonary embolism, hypoxia, pleural effusion, hypotension, diarrhea, urinary tract infections	[174]
Dabrafenib	treatment with trametinib in patients with locally advanced or metastatic BRAF V600E–mutated ATC.	BRAF inhibitor	150 mg/day	ORR: 69%	BRAF	fatigue (38%), pyrexia (37%), nausea (35%)	[177]
Trametinib	treatment with dabrafenib in patients with locally advanced or metastatic BRAF V600E–mutated ATC.	MEK inhibitor	2 mg/day	ORR: 69%	MEK1, MEK2	fatigue (38%), pyrexia (37%), nausea (35%)	[177]

ORR: overall response rate. PFS: progression-free survival.

### 6.2. Advances in Clinical Drug Research of Thyroid Therapy

In the past two decades, breakthroughs have been made in the study of the mechanism of aggressive TC, and many clinical trials related to targeted therapy have been conducted. This section will discuss some of the drugs in clinical trials, including their target sites, experimental phases, doses, effects and common adverse effects (Table 2).

Pazopanib can inhibit angiogenesis by inhibiting VEGFR, PDGFR, and c-Kit signaling [181]. In a phase II study, the median PFS and overall survival of RR-DTC patients in the experimental group were 11.4 and 2.6 months, respectively. The partial remission rate was 36.7%, and Pazopanib shows high clinical activity in patients with RR-DTC [182]. Additionally, another study on pazopanib revealed that intermittent treatment did not demonstrate obvious superiority to continuous treatment in terms of efficacy or tolerability [183]. Anlotinib is another drug that targets VEGFR, FGFR, PDGFR, and c-Kit and which inhibits angiogenesis and cell multiplication [184,185]. Compared with the control group, Anlotinib-treated patients with advanced thyroid cancer had an ORR of 76.9%. Anlotinib shows significant antitumor activity [186]. As a BRAF mutation inhibitor [187], in a study with seven subjects, the combination of vemurafenib and CDX-3379 was found to enhance the safety and efficacy of RAI uptake [188].

Apatinib, Surufatinib, and Donafenib all can inhibit VEGFR activity. Apatinib inhibits the kinase activity of VEGFR2 and cellular phosphorylation [189]. In comparison with the controlled trial with placebo, PFS lasted substantially longer and was safer in the experimental group [190]. Moreover, Surufatinib can target VEGFR1, VEGFR2, VEGFR3, FGFR1, and CSF1R [191]. An experiment of 77 patients demonstrated that Surufatinib has better efficacy and is safer for patients with locally terminal or metastatic MTC, RR-DTC, or locally terminal unresectable recurrences who cannot receive RAI [192]. Donafenib inhibits RAF phosphorylation and cuts off VEGFR and PDGFR signaling [193]. In a randomized multicenter phase II trial, patients with RR-DTC treated with Donafenib exhibited improved efficacy and safety [194].

Selumetinib can inhibit MEK1 and MEK2 [195]. In the phase III randomized controlled trial, there was no statistically significant difference between the experimental and control groups in terms of CR values among patients with DTC [196]. In this way, the drug dose needs to be reconsidered. Lenvatinib has been approved for treating patients with RR-DTC [162]. Nonetheless, the efficacy of lenvatinib against unresectable ATC is unsatisfactory because of the low survival rate and high incidence of adverse reactions. Lenvatinib’s efficacy in ATC patients must be explored more through additional studies [88].

**Table 2:** Ongoing clinical trials with anti-thyroid carcinoma drug.

Drug	Mechanism of Action: Target(s)	Stage of Development	Dosage	N	PR	PFS (Months)	Common Adverse Events	Trial Identifier	References
Pazopanib	VEGFR, PDGFR, c-kit	II	600 mg	60	36.7%	11.4	hypertension (21.7%), fatigue (8.3%), neutropenia (8.3%)	NCT00625846	[182]
Anlotinib	VEGFR, PDGFR, GFR, c-Kit	IIB	12 mg	91	48.4%	20.7	palmar–plantar erythrodysesthesia syndrome (62.9%), proteinuria (61.3%), hypertriglyceridemia (48.4%), hypertension (46.8%), diarrhea (40.3%)	NCT02586350	[186]
vemurafenib	BRAF	I	960 mg	7	N/A	N/A	maculopapular rash, diarrhea, arthralgia, HFS, nausea, alkaline phosphatase elevation	NCT02456701	[188]
Apatinib	VEGFR2	III	500 mg	92	54.3%	22.2	hypertension (34.8%), palmar plantar syndrome (17.4%), proteinuria(15.2%), diarrhea (15.2%)	NCT03048877	[190]
Surufatinib	VEGFR1, VEGFR2, VEGFR3, FGFR1, CSF1R	II	300 mg	59	23.2%	11.1	hypertension (20.3%), proteinuria (11.9%), elevated blood pressure (5.1%), hypertriglyceridemia (5.1%), pulmonary inflammation (5.1%)	NCT02614495	[192]
Donafenib	VEGFR, PDGFR	II	200 mg 300 mg	35	200 mg 12.5% 300 mg 13.33%	200 mg 9.44 300 mg 14.98	palmar plantar syndrome (82.86%), alopecia (71.43%), hypertension (45.71%)	NCT02870569	[194]
Selumetinib	MEK1, MEK2	III	75 mg	233	CR 40%	18	dermatitis acneiform (45%), diarrhea (44%), fatigue (29%), nausea (29%), edema peripheral (19%), pruritus (14%), hypertension (13%), rash maculopapular (12%), stomatitis(11%)	NCT01843062	[196]
lenvatinib	VEGF1, VEGF2, VEGF3, FGF1, FGF2, FGF3, FGF4, PDGFα, RET, KIT	II	24 mg	42	9.5%	7.4	loss of appetite (48.0%), fatigue (48.0%), hypertension (44.0%), palmar–plantar erythrodysesthesia syndrome (26.0%)	NCT02726503	[88]

PR: Partial Response. PFS: Progression-Free Survival.
